# CRISPR–Cas adaptation in *Escherichia coli* requires RecBCD helicase but not nuclease activity, is independent of homologous recombination, and is antagonized by 5′ ssDNA exonucleases

**DOI:** 10.1093/nar/gky799

**Published:** 2018-09-05

**Authors:** Marin Radovčić, Tom Killelea, Ekaterina Savitskaya, Lukas Wettstein, Edward L Bolt, Ivana Ivančić-Baće

**Affiliations:** 1 Department of Biology, Faculty of Science, University of Zagreb, Croatia; 2 School of Life Sciences, University of Nottingham, UK; 3 Center for Life Sciences, Skolkovo Institute of Science and Technology, Moscow 143028, Russia; 4 Institute of Molecular Genetics, Russian Academy of Sciences, Moscow 123182, Russia

## Abstract

Prokaryotic adaptive immunity is established against mobile genetic elements (MGEs) by ‘naïve adaptation’ when DNA fragments from a newly encountered MGE are integrated into CRISPR–Cas systems. In *Escherichia coli*, DNA integration catalyzed by Cas1–Cas2 integrase is well understood in mechanistic and structural detail but much less is known about events prior to integration that generate DNA for capture by Cas1–Cas2. Naïve adaptation in *E. coli* is thought to depend on the DNA helicase-nuclease RecBCD for generating DNA fragments for capture by Cas1–Cas2. The genetics presented here show that naïve adaptation does not require RecBCD nuclease activity but that helicase activity may be important. RecA loading by RecBCD inhibits adaptation explaining previously observed adaptation phenotypes that implicated RecBCD nuclease activity. Genetic analysis of other *E. coli* nucleases and naïve adaptation revealed that 5′ ssDNA tailed DNA molecules promote new spacer acquisition. We show that purified *E. coli* Cas1–Cas2 complex binds to and nicks 5′ ssDNA tailed duplexes and propose that *E. coli* Cas1–Cas2 nuclease activity on such DNA structures supports naïve adaptation.

## INTRODUCTION

CRISPR–Cas is a prokaryotic adaptive immune system against mobile genetic elements (MGEs) in bacteria and archaea (1,2). Immunity is acquired through capture of MGE DNA fragments (‘protospacers’) and their site-specific integration into a CRISPR array as ‘spacers’ positioned between repeat DNA sequences. These processes are called adaptation and are catalysed by Cas1–Cas2 integrase from host CRISPR–Cas systems aided by other host proteins, reviewed recently in (3). ‘Naïve adaptation’ relies on Cas1–Cas2 for cells to establish new immunity against an MGE that has not been previously encountered by integration of new spacer DNA into CRISPR arrays (4). Immunity is effected by transcription of the CRISPR array and transcript processing into shorter RNA molecules (crRNAs) that comprise a single spacer sequence. Assembly of crRNA into a ribonucleoprotein complex is used to recognize complementary MGE DNA ‘protospacer’ sequence by base pairing with crRNA, beginning processes of CRISPR ‘interference’. In *Escherichia coli*, interference R-loops are formed by Cascade (CRISPR-associated complex for antiviral defence) after detecting MGE DNA through a protospacer adjacent motif (PAM) sequence (5,6). Cascade R-loop formation recruits Cas3 nuclease/helicase for degradation of the MGE DNA thus completing the immunity response (7–9).

Adaptation processes that generate prokaryotic immunity to an MGE can be separated into three major stages: MGE DNA capture, transport to a CRISPR array, and DNA integration into the CRISPR array followed by DNA gap filling to duplicate the associated repeat (10). Cas1 and Cas2 proteins encoded within CRISPR–Cas systems catalyse these processes aided by other host cell nucleic acid processing proteins. In *E. coli*, there is substantial mechanistic detail known about how Cas1–Cas2 bound to MGE DNA recognizes CRISPR and subsequently integrates the DNA. A Cas1–Cas2 complex comprising Cas1 dimers held together by a Cas2 dimer is essential for adaptation in *E. coli* (11–13) binding to a short DNA duplex with flayed ssDNA ends in an adaptation ‘capture complex’ (11,14). The Cas1–Cas2 capture complex is guided to the CRISPR array by DNA structures formed by binding of *E. coli* integration host factor (IHF) to a conserved sequence motif within the promoter (‘leader’) sequence of CRISPR (15,16). The 3′OH groups of DNA in the capture complex direct nucleophilic attack of the CRISPR array catalysed by Cas1. This generates a half-site DNA intermediate from the first nucleophilic attack at the leader/promoter-end of CRISPR and then full site integration following the second nucleophilic attack at the repeat-spacer boundary (13,17–19). Host DNA repair gap-fills the integration site (20), completing adaptation by incorporation of a new spacer and new DNA repeat.

DNA pre-processing that leads to capture by Cas1–Cas2 is much less well understood than DNA integration. The Cas1–Cas2–DNA capture complex has been identified at the point of integration (17,19) but the genesis of DNA leading to capture is unclear. Pre-spacers should originate from MGE DNA, to avoid lethal autoimmunity, and their processing should be at specific position relative to PAM. Cas1 monomers contain a PAM-sensing region and Cas1 mediated processing of pre-spacers creates the 3′OH ends required for nucleophilic attack (12,14). Naïve adaptation requires active DNA replication or active transcription and majority of protospacers are non-randomly distributed with many acquired around the origin of replication (*oriC*), terminus (*ter*), CRISPR, rDNA loci, R-loops—specific regions known to experience DNA nicking or double-strand breaks. *E. coli* naïve adaptation is stimulated by RecBCD enzyme during the repair of double-stranded breaks (DSB) that may arise from stalled replication forks (21). RecBCD is thought to aid naïve adaptation by generating single-stranded DNA (ssDNA) intermediates from helicase and nuclease activities before reaching a *Chi* site (5′-GCTGGTGG-3′) that attenuates these activities. In this model ssDNA generated by RecBCD nuclease re-anneals into partial duplex that is a substrate for Cas1–Cas2 (21). During naïve adaptation integration of host fragments as new spacers occurs but spacer integration from a plasmid MGE is more frequent (21–23). The frequency of new MGE DNA spacers derived from the *E. coli* chromosome were ∼10-fold higher in *recB, recC* and *recD* mutants compared to the *wt* strain suggesting that RecBCD also helps in self/non-self discrimination, or that DNA substrates generated in these mutant backgrounds are particular targets for capture during adaptation. In this work, we analysed involvement of RecBCD and other host nucleases in naïve adaptation using genetic analysis. This indicated that (a) nuclease activity of RecBCD is not required for adaptation, (b) helicase, or other, activity of RecBCD promotes adaptation and (c) recombination by RecA that is stimulated by RecBCD inhibits adaptation. We also show that purified Cas1–Cas2 complex can act as a nuclease with specificity for a 5′ ssDNA tailed duplexes, substrates that genetics implied are important for stimulating adaptation.

## MATERIALS AND METHODS

### Strains, plasmids, media and general methods

*Escherichia coli* strains used are described in Supplementary Table S1. Mutant bacterial strains were made by P1 *vir* transduction and selected for the appropriate antibiotic resistance. Antibiotic resistance genes were eliminated using pCP20 (24). Bacteria were grown at 37°C in LB broth (10 g/l bacto-tryptone, 5 g/l yeast extract, 10 g/l NaCl) and on LB agar plates (supplemented with 15 g of agar for solid media). When required appropriate antibiotics were added to LB plates at final concentrations: ampicillin at 100 μg/ml, kanamycin at 40 μg/ml, apramycin 30 μg/ml, tetracycline 10 μg/ml, spectinomycin 100 μg/ml, trimethoprim 100 μg/ml and chloramphenicol at 15 μg/ml. Plasmids used were pBad-HisA (Invitrogen) as an empty plasmid vector control and pEB628 for arabinose inducible expression of Cas1–Cas2 from pBad-HisA described in (20).

### Naïve adaptation assay and plasmid instability

New spacer acquisition into a CRISPR locus by naïve adaptation was assessed by the procedure described in (4,20,25). Cells lacking chromosomally encoded Cas3, Cascade and Cas1–Cas2 were transformed by pEB628 (pCas1–Cas2) or pBad-HisA and individual transformants were inoculated in LB broth. Expression of Cas1–Cas2 was induced by addition of 0.2% (w/v) l-arabinose. Cells were aerated at 37°C for 16 h and then sub-cultured (‘passaged’) up to three times by diluting 1:300 the previous overnight culture into fresh LB with arabinose. Spacer acquisition was monitored by PCR using primers detailed in (20) followed by agarose gel electrophoresis on 2% agarose gels stained using SYBR safe. Template DNA was prepared from bacterial cultures by boiling in water. Relative band intensities for spacer acquisition quantification were measured using Kodak 1D Image Analysis Software v. 3.6.0. This software detected bands containing no spacer automatically, while the spacer containing bands were manually marked by a rectangle. The rectangle was used to mark all of the bands, including the bands of the negative control lanes, i.e. the PCR products of strains transformed with the empty vector pBad. In this way, the relative intensity values of bands were calculated by subtracting values with pBad from the corresponding values of the same strain with pCas1–Cas2. At least two independent experiments were done for each strain.

Each passage of naïve adaptation was also analysed for instability of pBad or pEB628 by viability ‘spot’ tests of cell survival on ampicillin agar. Cells were serially diluted in 67 mM phosphate buffer (pH 7.0) and 10 μl aliquots were spotted onto LB and LB with ampicillin plates for incubation overnight at 37°C. Cells having lost the plasmid gave lower viable counts on ampicillin plates in comparison to LB plates. We also studied the plasmid presence in cells grown to log phase (OD_600_ = 0.5) in the presence of l-arabinose and antibiotic ampicillin. Cells were also serially diluted and analysed as above.

### Spacer acquisition analysis and mapping

Spacer aquisition experiments for strains IIB1165 (*wt*), IIB1214 (*recB1080*) and IIB1245 (*recD recA)* were assessed from cells grown as described above. Cells were ‘passaged’ two times for strains IIB1165 and IIB1245 and only once for IIB1214 (two biological replicas). PCR products that correspond to expanded CRISPR array were gel purified with Promega Wizard SV Gel and PCR Clean Up System. Sequencing was performed on Illumina Miniseq platform in 2 × 150 paired end mode. R packages ShortRead and BioString were utilized for reads pre-processing and downstream analysis, mapping and mapping visualization. During pre-processing reads with Phred quality score of <20 were trimmed, and reads with two or more CRISPR repeats were filtered. Sequences between two CRISPR repeats determined with two mismatches allowed were extracted as spacers. Spacers were mapped first to the plasmid (unique mapping for plasmid locations) and those that did not match the plasmid were mapped to the genome, non-unique matches were discarded. Disregarding quantities (every spacer counts only once) were applied for statistical analysis of spacer distribution.

### Protein purification

Cas1 and Cas2 proteins were over-expressed individually according to the method described in (20) generating Cas1 with an N-terminal (His)_6_-tag and untagged Cas2. Cell biomass for over-expression was thawed, sonicated and clarified. The resulting lysates were combined and mixed for 2 hours at 4°C. This allows purification of stable Cas1–Cas2 complex that is identifiable in gel filtration and elutes separately from either Cas1 or Cas2 alone (Supplementary Figure S1A), and which is active *in vitro* for catalysing half- and full-site integration of duplex DNA into a CRISPR locus (Supplementary Figure S1B). Cas1- Cas2 was bound to a 5 ml HiTrap Chelating column (GE Healthcare) charged with nickel. Unbound protein was washed with buffer A (20 mM Tris pH 7.5, 500 mM NaCl, 20 mM imidazole, 10% glycerol) with bound protein eluted using a linear gradient of 20–500 mM imidazole over 25 ml. Following dialysis in buffer B (20 mM Tris pH 7.5, 150 mM NaCl, 1 mM DTT, 10% glycerol) Cas1–Cas2 was further purified using a 1 ml HiTrap Heparin HP column (GE Healthcare), washed with buffer B and eluted using a linear gradient 150 mM–1 M NaCl. Separation of Cas1–Cas2 from unbound Cas1 was achieved by elution from an Superdex 200 Increase 10/300 GL (GE Healthcare) using buffer C (20 mM Tris pH 7.5, 150 mM KCl, 20% glycerol, 1 mM DTT) prior to storage at –80°C.

Genes encoding *E. coli* IHF α and β subunits were PCR amplified using the primers listed in supplementary data for cloning into pACYCduet using sites for restriction endonucleases BamHI/NotI and XhoI/AvrII respectively. Co-expression of IHF subunits was in *E. coli* BL21AI cells grown at 37°C to O.D._600_ of 0.6 followed by induction with 0.2% l-arabinose and 0.5 mM IPTG with growth continued overnight at 18°C. Harvested cells were resuspended in buffer J (500 mM KCl, 20 mM HEPES pH 7.5, 20 mM imidazole, 0.1% Triton X-100, 10% glycerol) plus 1× protease inhibitor cocktail tablet (EDTA free) (Roche). IHF subunits were co-purified using a 5 ml HiTrap Chelating column (GE Healthcare) charged with nickel. Unbound protein was washed with buffer J and bound protein eluted in an isocratic elution buffer J plus 500 mM imidazole. Eluted protein was dialysed overnight at 4°C in buffer K (150 mM KCl, 20 mM HEPES pH 7.5, 0.1% Triton X-100, 10% glycerol), followed by further purification using a 1 ml HiTrap Heparin HP column (GE Healthcare), washed with buffer K and eluted using a linear gradient of 150 mM–1 M KCl. Fractions containing both subunits were pooled and flash frozen for storage at –80°C.

### DNA substrates and Cas1–Cas2 EMSA and DNA nicking assays

Sequences of DNA oligonucleotides and the substrates generated for this work are presented in Supplementary Figure S2. Substrates were 5′-Cy5-end labelled for visualization in gels. EMSAs to assess binding of Cas1–Cas2 to tailed duplex DNA molecules were in 5% acrylamide TBE gels, after mixing at 37°C for 30 min Cas1–Cas2 and DNA (20 nM) in buffer HB (20 mM Tris–HCl pH 8.0, 100 μ/ml bovine serum albumin, 7% glycerol) and loaded directly onto the gels. Gels were electrophoresed for 1.5 h at 120 V. DNA cutting activity of Cas1–Cas2 was assessed in 15% TBE gels containing 8 M urea. Cas1–Cas2 was mixed with 20 nM DNA and buffer HB with addition of magnesium chloride (10 mM) for incubation at 37°C for 60 min. Reactions were stopped by adding proteinase K and EDTA for loading heated samples onto urea gels in formamide loading buffer.

## RESULTS

### Genetic analysis of RecBCD nuclease activity in naïve adaptation

**Figure 1.**
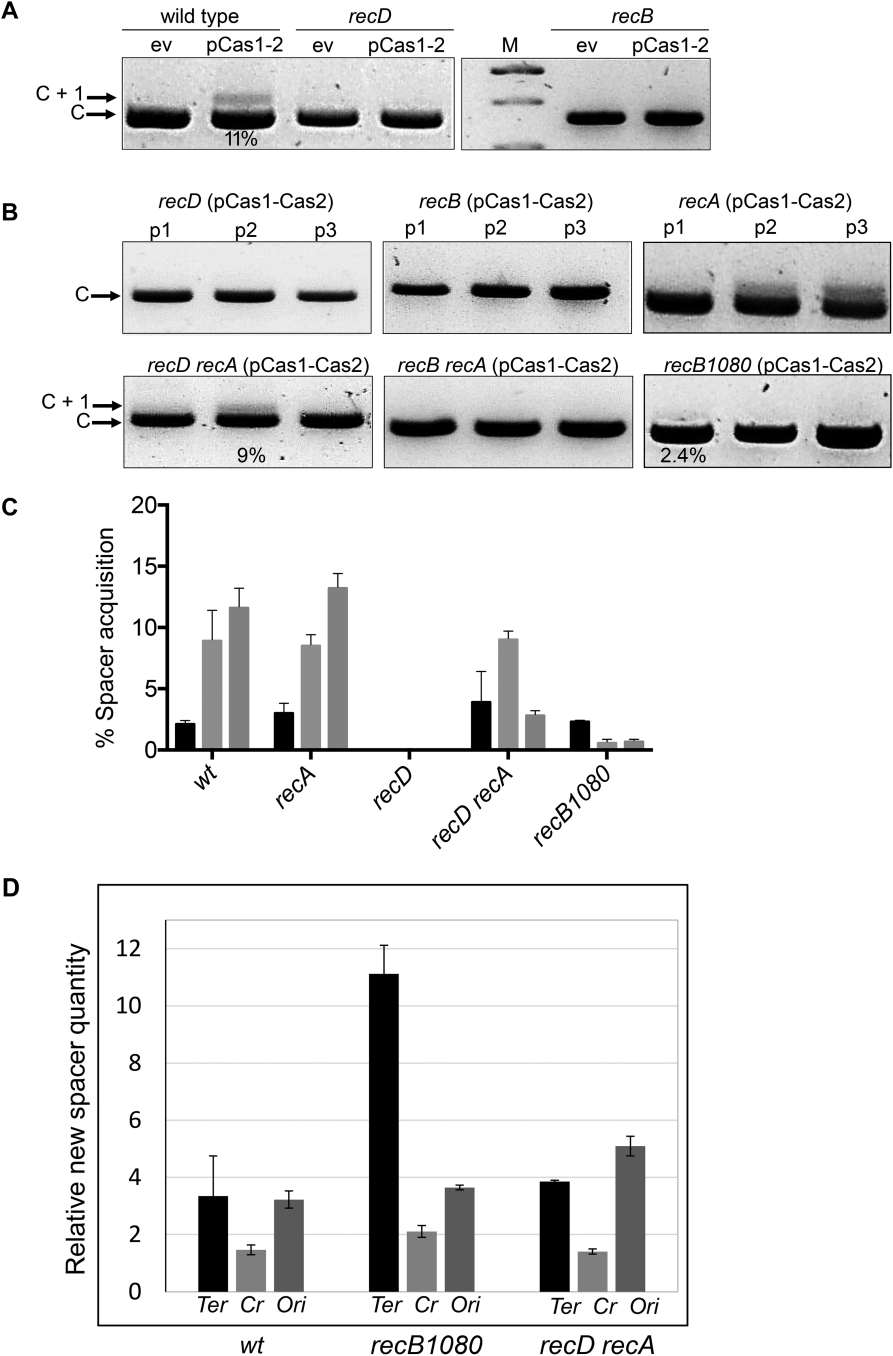
Genetic analysis of RecBCD in naïve adaptation. (**A**) Agarose gels summarizing PCR-based detection of *E. coli* CRISPR-1 expansion after integration of a new spacer (C+1) during naïve adaptation. Strains are indicated above each panel (wt, wild type) as are plasmids either pBad-HisA (ev, empty vector) or pEB628 for arabinose inducible Cas1–Cas2 (pCas1–2). Results from the third passage are presented. (**B**) Agarose gels summarizing CRISPR expansion in the *E. coli* strains indicated in three passages (p1 – p3) in all cases containing plasmid encoding inducible Cas1–Cas2 (pCas1–2). (**C**) Measurements of new spacer acquisition detectable as expansion of CRISPR-1 (C+1) using PCR of chromosomal DNA from the strains indicated. See also Supplementary Table S3. Percentage spacer acquisition refers to intensity of C +1 DNA/(C+1 DNA + C DNA). Each strain indicated below the x-axis has three histograms representing measured adaptation in passage one (black), two (light grey) and three (light grey). (**D**) The relative quantities of spacers mapped to specified chromosomal regions. The spacers mapped onto 670 kb area spanning either Terminus (Ter) regions, CRISPR arrays (Cr) or Origin (Ori) regions were added and normalized to the number of spacers mapped to the *E. coli* chromosomal region spanning 0–670 kb.

In current models of naïve adaptation in *E. coli* RecBCD nuclease activities that promote DNA repair by homologous recombination also generate DNA for capture by Cas1–Cas2, leading to adaptation (21). In previous work (20) it was demonstrated that *recB* was required for wild-type levels of naïve adaptation but *recA* was not, indicating that naïve adaptation is independent of RecA catalyzed recombination. To better understand this, given that a major role for RecBCD in DNA repair is to load RecA, we carried out detailed genetic analysis using multiple alleles of RecBCD and assessed naïve adaptation. Naïve adaptation was detected by expansion of the CRISPR-1 locus in an *E. coli* K-12 strain that lacks functioning chromosomal Cas proteins (Supplementary Table S1) but has the chromosomal CRISPR-1 locus and expresses Cas1 and Cas2 from an inducible plasmid, summarized in Figure 1A. Acquisition of new spacer DNA was clearly visible in wild type cells after three passages of growth.

Compared with wild type *E. coli* cells, naïve adaptation was severely reduced or undetectable in cells inactivated for *recD* or *recB* in end point assays (Figure 1A) or when tested over three growth passages (Figure 1B and additional data in Supplementary Results Table S2). These results are in agreement with a model in which RecBCD nuclease activity is important for naive adaptation in *E. coli* (21) because neither *recB* or *recD* cells possess RecBCD nuclease activity. However, two further genetic traits of *recB* and *recD* cells were assessed, the effect of RecA loading onto DNA and plasmid stability, because they potentially impact on naive adaptation.

RecBC enzyme in cells inactivated for *recD* is a nuclease-free helicase that constitutively loads RecA onto 3′ ssDNA to initiate recombination (26). We observed that naïve adaptation was restored to measurable levels similar to wild type when *recA* was also removed to generate a *recD recA* double mutant background (Figure 1B and C and Supplementary Table S3). As established in previous work (20), deletion of *recA* alone has no discernable effect on naïve adaptation. Interestingly, in these assays naïve adaptation was not readily restored to *recB recA* cells (Figure 1B) that lack both RecBCD nuclease and helicase activity. Analysis of adaptation in *recB recA* cells using further iteration of PCR did detect some new spacer product but at significantly reduced efficiency compared to wild type cells (Supplementary Table S2). However, reduced adaptation associated with *recB recA* cells suggested that helicase activity, unlike nuclease activity, of RecBCD does promote naïve adaptation. Analysis of naïve adaptation in cells carrying the RecBCD allele *recB1080* further supported that RecBCD nuclease activity is dispensable for naïve adaptation (Figure 1C). This mutation encodes RecB^1080A^CD protein that lacks nuclease activity and RecA loading, but helicase activity is retained (27,28). Spacer acquisition in *recB1080* cells after a single passage was comparable to wild type cells (Figure 1C and Supplementary Table S3) but dropped away in passages two and three due to plasmid instability compared to wild type cells (Supplementary Table S4). In summary, the genetic analyses indicate that cells lacking RecBCD nuclease activity are proficient at naïve adaptation.

These assays for naïve adaptation were measured over three passages to account for plasmid instability that is associated with *recBCD* mutations in *E. coli* (29). Elimination of the Cas1–Cas2 plasmid results in loss of adaptation over time in these genetic backgrounds, for example as was observed in the third passage of *recD recA* cells (Figure 1B and C and Supplementary Table S3). Full measurements of plasmid instability correlating to adaptation are presented in Supplementary Table S4. It is significant that naïve adaptation in *recD recA* cells was readily detectable in passage 2 even though instability of plasmid expressing Cas1–Cas2 resulted in its loss with >200 – fold greater frequency compared to in wild type cells (Supplementary Table S4).

High throughput sequencing of DNA in extended CRISPR arrays identified that newly acquired spacers mapped to plasmid and genomic DNA and that no strand bias was detected, as expected for naive adaptation. Our analysis identified that most spacers (79–90%) originated from the *E. coli* chromosome in wild type and RecBCD/RecA mutant strains compared to acquistion from plasmid pEB628 that was used for expression of Cas1–Cas2 (Supplementary Figure S3A). Close examination of the pattern of spacer mapping onto the chromsome highlighted that in all cells analyzed 3–4 times more newly acquired spacers originated from origin (*ori*) and termination (*ter*) regions of the chromosome relative to the reference genomic region spanning the same distance (670 kb, Figure 1D). *recB1080* cells were associated with >10 times more new spacers being acquired from *ter* sites, an effect not observed for *recD recA* cells (Figure 1D). These observations might be explained by loss of RecBCD functionality triggering accumulation of aberrant or unprocessed intermediate DNA structures arising during replication termination or recombination (29–31). Information for accessing raw DNA sequencing data underlying these results is given at the end of this manuscript.

### The effect of exonucleases on naïve adaptation in *E. coli*

**Figure 2.**
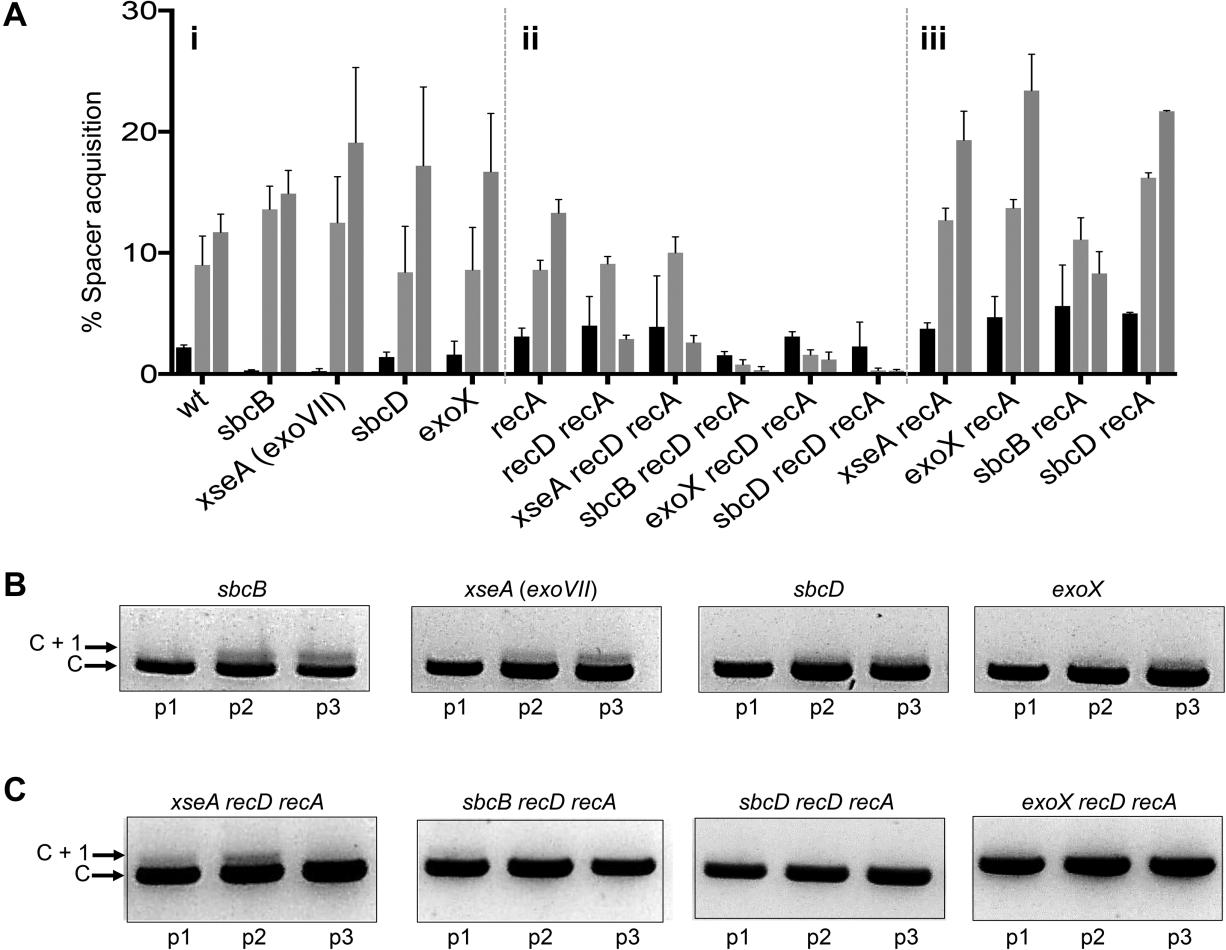
Analysis of 3′→5′ ssDNA exonucleases in naïve adaptation. (**A**) Graph summarizing measurements of new spacer acquisition in the strains indicated detectable as expansion of CRISPR-1 (C+1) using PCR of chromosomal DNA from the strains indicated. See also Supplementary Table S3. Percentage spacer acquisition refers to intensity of C +1 DNA/(C+1 DNA + C DNA). Each strain indicated below the x-axis has three histograms representing measured adaptation in passage one (black), two (light grey) and three (light grey). (**B** and **C**) Agarose gel slices summarizing naïve adaptation effects shown for strains selected from the graph. All strains contained the plasmid encoding inducible Cas1–Cas2 complex (pCas1–2).

We investigated if naïve adaptation was supported by nucleases other than RecBCD by testing if new spacer acquisition was affected by inactivating *E. coli* exonucleases that promote genome stability (32,33). Inactivation of individual 3′ to 5′ ssDNA exonucleases SbcB (also called ExoI), ExoVII (XseA subunit of XseAB complex), SbcCD or ExoX did not impinge on adaptation over three passages (Figure 2Ai, B and Supplementary Table S3) and combining these with inactivation of *recD* deletion gave cells that remained unable to acquire new spacers like the *recD* deletion alone (Supplementary Figure S3B). Restoration of adaptation in *recD recA* cells (Figure 1) was used to assess if any of the 3′ to 5′ ssDNA nucleases are required for adaptation, which would manifest as reduced spacer acquisition by inactivating the nuclease in *recD recA* cells. Deletion of *xseA* (*exoVII*) in *recD recA* cells had little effect on adaptation over three passages compared to *recD recA* cells (Figure 2Aii and Supplementary Table S3) and plasmid instabilities associated with these strains were similar (Supplementary Table S4), indicating no effect of *xseA* in this context. Deletion of *sbcB, sbcD* or *exoX* in *recD recA* cells all gave significantly reduced adaptation compared to *recD recA* cells in all passages (Figure 2Aii and C), but this correlated to 10-fold increased plasmid instability (Supplementary Table S4). Therefore, it is likely that reduced adaptation by inactivation of these nucleases is caused by loss of Cas1–Cas2 encoding plasmids in these assays. To determine if these exonucleases are required for adaptation when RecBCD enzyme is functional we inactivated them in combination with the *recA* mutation only. Adaptation was not affected in *sbcD recA, exoX recA* or *sbcB recA* cells compared to wild type cells (Figure 2Aiii), and these cells showed much improved plasmid stability (Supplementary Table S4). Overall these results indicate that naïve adaptation does not require these 3′ ssDNA exonucleases.

**Figure 3.**
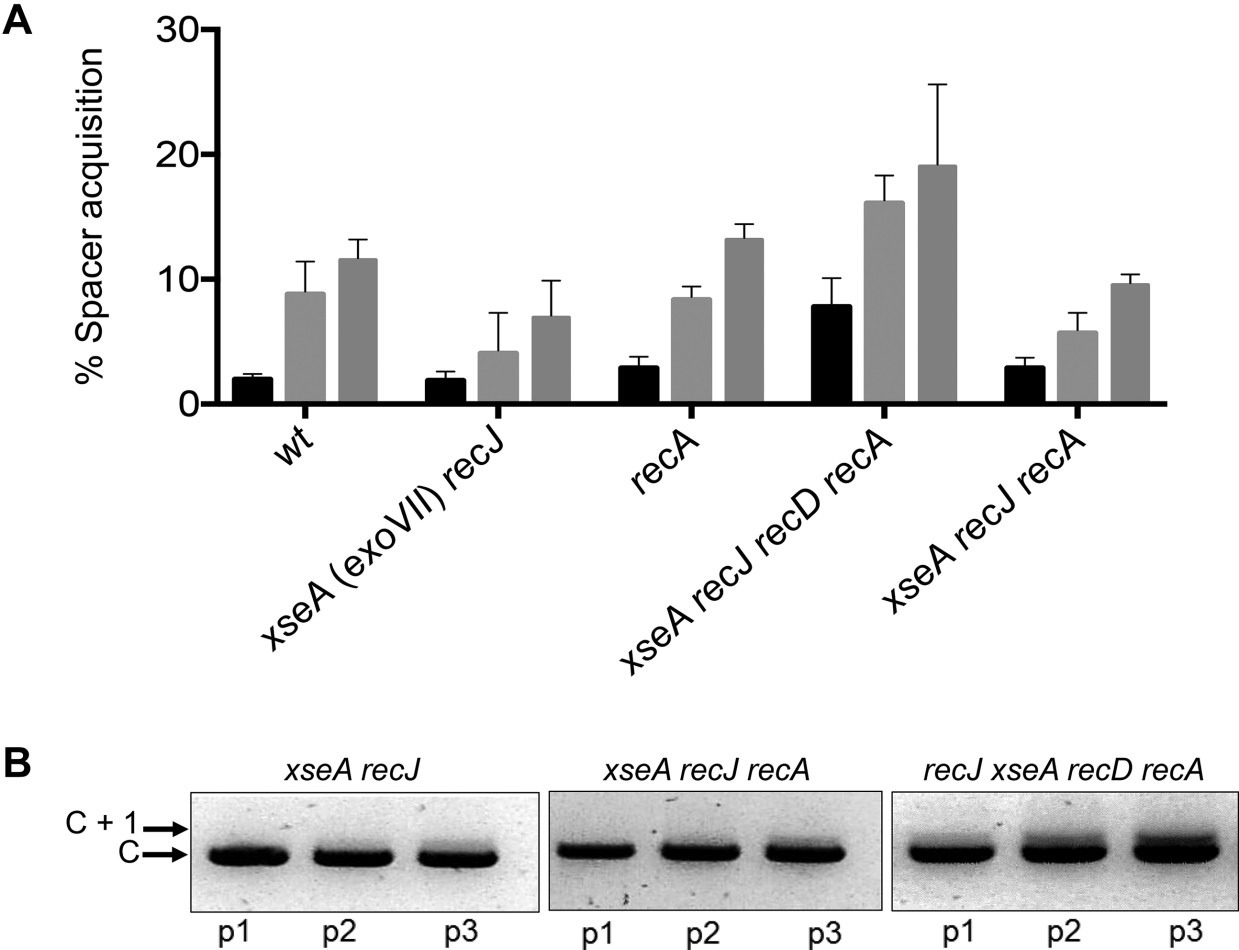
Analysis of 5′→3′ ssDNA exonucleases in naïve adaptation. (**A**) Graph summarizing measurements of new spacer acquisition in the strains indicated detectable as expansion of CRISPR-1 (C+1) using PCR of chromosomal DNA from the strains indicated. See also Supplementary Table S3. Percentage spacer acquisition refers to intensity of C +1 DNA/(C+1 DNA + C DNA). Each strain indicated below the x-axis has three histograms representing measured adaptation in passage one (black), two (light gray) and three (light gray). (**B**) Agarose gels summarizing CRISPR expansion in the *E. coli* strains indicated in three passages (p1–p3) in all cases containing plasmid encoding inducible Cas1–Cas2 (pCas1–2).

We investigated if 5′ to 3′ ssDNA exonuclease activities of RecJ and ExoVII (encoded by *xseAB*) influence naïve adaptation in *E. coli*. Adaptation was proficient after inactivation of *recJ* or *xseA* or both (Figure 3A and Supplementary Table S3) but could not be detected in *recD recJ/xseA* cells, as expected because of the dominant negative effect of the *recD* mutation (Supplementary Figure S3C). In contrast to results from the 3′ ssDNA exonucleases, when *recA recD* cells were used to unmask any effect on adaptation of 5′ to 3′ exonucleases we observed that inactivation of *recJ* and *xseA (xseA recJ recD recA* cells) significantly increased new spacer acquisition compared to wild type and *xseA recJ recA* cells (Figure 3). This suggested that functioning RecJ and ExoVII have a negative effect on naïve adaptation that is alleviated by removing them, implying that DNA molecules with 5′ ssDNA tails stimulate naïve adaptation.

### Cas1–Cas2 complex binds to and nicks 5′-tailed partial duplexes

**Figure 4.**
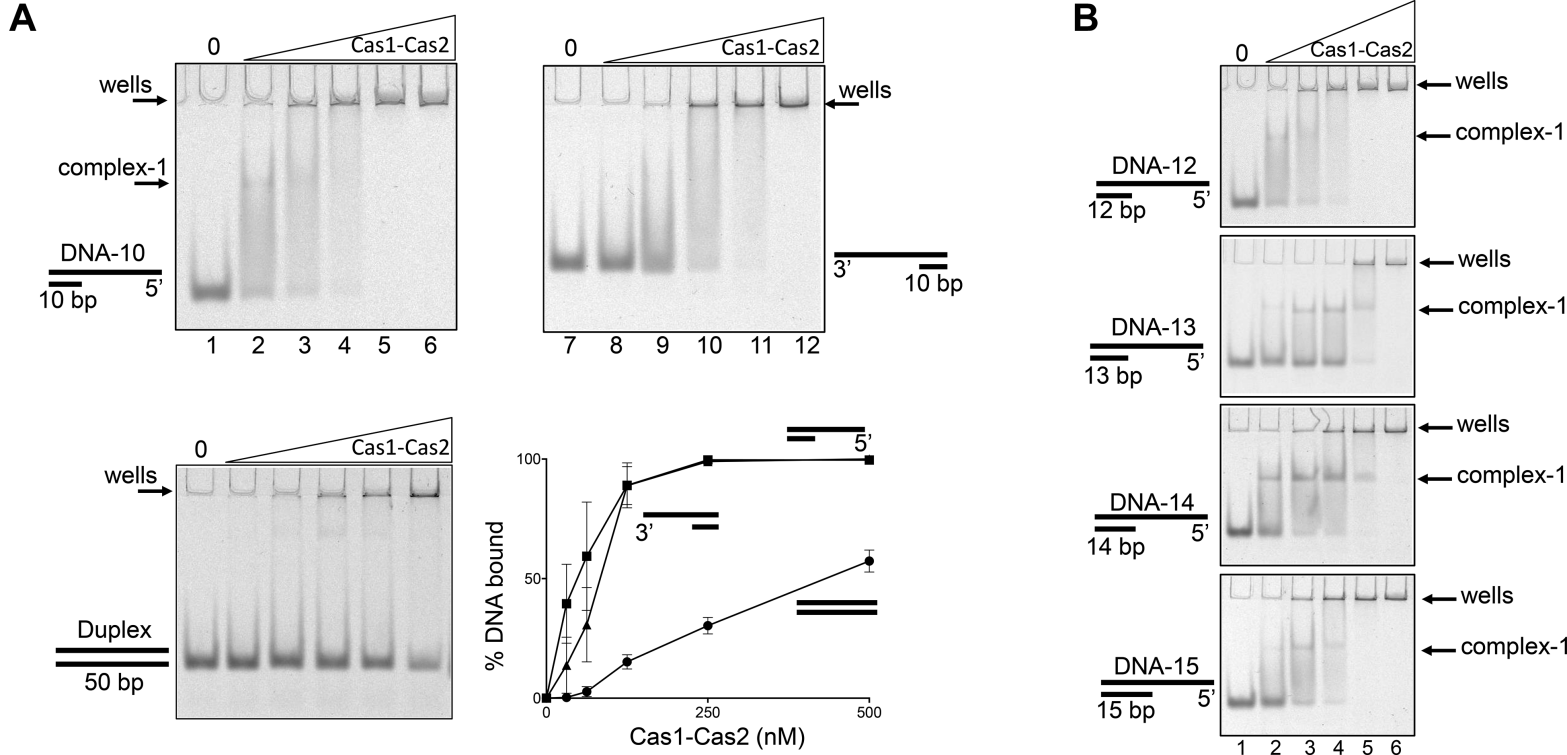
Comparative mobility shift analysis of Cas1–Cas2 binding to DNA substrates. (**A**) Electrophoretic mobility shift analysis of increasing concentrations of Cas1–Cas2 binding to 5′ overhang, 3′ overhang and duplex DNA as indicated. Oligonucleotide sequences used to prepare the substrates are shown in Supplementary Figure S2. Cy5 end labeled DNA substrates (20 nM) were incubated with 0, 31.25, 62.5, 125, 250, 500 nM Cas1–Cas2 complex for 30 min at 37°C followed by analysis on a 5% native acrylamide gel and imaged using a FLA3000 (FujiFilm). The graph shows quantified binding of Cas1–2 to 5′ overhang (▪), 3′ overhang (▴) and duplex DNA (•) DNA substrates. Band quantification was carried out using ImageJ (NIH) as a normalized value of bound substrate as a percentage of total Cy5 fluorescence per lane, with error bars showing the standard error (*n* = 3). (**B**) EMSAs comparing Cas1–Cas2 complex formation with 5′ssDNA tailed duplexes of varying lengths, as indicated. Assay conditions were the same as used in EMSAs in part A.

**Figure 5.**
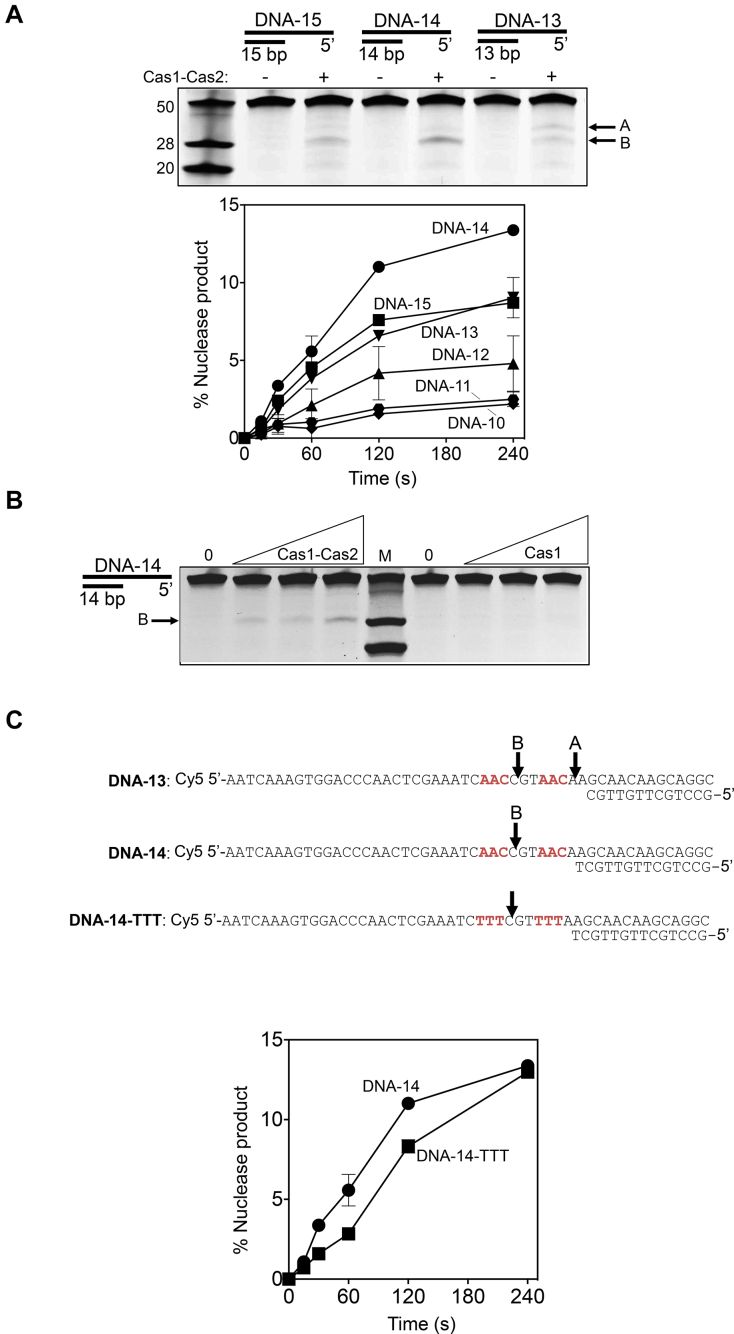
Nicking of DNA substrates by purified Cas1–Cas2 complex. (**A**) A summary of Cas1–Cas2 nicking activity on 5′-ssDNA tailed DNA duplexes, as indicated. Oligonucleotide sequences used to prepare the substrates are shown in Supplementary Figure S2. Marker ssDNA nucleotide lengths are given to the left of the gel panel. Cy5 end labeled DNA substrates (20 nM) were incubated with 0 or 250 nM Cas1–Cas2 complex for 60 minutes at 37°C, followed by analysis on a 15% denaturing acrylamide gel and imaged using a FLA3000 (FujiFilm). Arrows indicate the major nicking products (A and B) generated by Cas1–Cas2. The graph shows cutting activity of Cas1–Cas2 complex (250 nM of total protein) on 5′-ssDNA tailed DNA duplexes (20 nM) as indicated, as a function of time. Reactions were in duplicate and error bars represent standard deviation from the mean values. Details of each substrate are given in Supplementary Figure S2. (**B**) Nuclease activity on DNA-14 (20 nM) of Cas1–Cas2 complex (0, 62.5, 125 and 250 nM) compared to the same assays containing only Cas1 at the same concentrations. The three DNA marker fragments are the same as Figure 5A and the major cutting product B is indicated. (**C**) Illustration of Cas1–Cas2 cutting sites identified in substrates (see also Supplementary Figure S6). The graph compares Cas1–Cas2 (250 nM) cutting activity, as a function of time, when mixed with DNA-14 and DNA-14-TTT, as indicated, and is plotted as means of two independent assays with standard deviation displayed as error bars.

Genetic analyses implied that DNA duplexes with 5′ ssDNA tails promote naïve adaptation. We used purified *E. coli* Cas1–Cas2 complex (Supplementary Figure S1A) that is proficient in catalyzing new spacer integration *in vitro* (Supplementary Figure S1B), for investigating binding and processing of ssDNA tailed substrates in potential DNA capture events (Figures 4 and 5). Previous work showed that Cas1–Cas2 stably bound to fork and other branched DNA molecules that might be explained by their resemblance to half-site intermediates formed during Cas1–Cas2 catalyzed integration reactions but which may not be relevant to DNA capture (20). Cas1–Cas2 binding and catalysis was therefore assessed on duplex DNA molecules with ssDNA tails that cannot undergo spacer integration reactions.

Cas1–Cas2 bound to 3′- and 5′-ssDNA tailed molecules with 10-base-pair duplex regions and 40 nucleotides of ssDNA, but not to a corresponding fully base-paired duplex (Figure 4A). Binding of Cas1–Cas2 to tailed duplexes in these EMSAs included significant protein-DNA aggregation in gel wells, but a stable protein-DNA complex could be discerned from binding to the 5′-ssDNA tailed 10 bp duplex (‘DNA-10’) in addition to protein aggregates (Complex-1 in Figure 4A lanes 2 and 3). This Cas1–Cas2 binding pattern with DNA-10 was also seen in control reactions binding Cas1–Cas2 to a duplex DNA that was previously optimised for productive integration reactions (Supplementary Figure S4)((11,12)). However, Cas1–Cas2 complex formation in EMSAs was significantly improved by increasing the length of the duplex region of the 5′ ssDNA tailed duplexes to 14 base pairs (Figure 4B, ‘DNA-14’).

Interestingly, Cas1–Cas2 cut the DNA backbone in the same 5′ ssDNA substrates that were bound in EMSAs, summarised in Figure 5A for substrates DNA-13, -14 and -15 that gave maximal activity of Cas1–Cas2 (up to 14% of DNA cut). Cas1 protein alone did not cut DNA-14, on which Cas1–Cas2 was most active (Figure 5B) indicating that active adaptation ‘capture complex’ (12) is needed for DNA cutting. The equivalent 3′ ssDNA substrate was not cut by Cas1–Cas2 complex (Supplementary Figure S5). Major products of Cas1–Cas2 DNA cutting DNA-10, -13, -14 or -15 (products A and B) were mapped to within ssDNA one nucleotide from AAC sequence (Figure 5B and Supplementary Figure S6), which is recognized as an *E. coli* PAM (34). To determine if this sequence was prerequisite for DNA cutting by Cas1–Cas2 we altered it to TTT in DNA-14, but this had little effect on product formation (Figure 5C). The results suggest that DNA structure (ssDNA and position of cut site relative to duplex DNA) may be important dictating efficacy of DNA cutting in these substrates. The *in vitro* activity of purified Cas1–Cas2 complex is compatible with observation from genetics that 5′ ssDNA tailed duplexes are important as substrates for adaptation and may be bound and cut by Cas1–Cas2 for DNA capture.

## DISCUSSION

CRISPR–Cas immunity in *E. coli* is established by naïve adaptation that involves capture of DNA fragments for integration into CRISPR loci by the Cas1–Cas2 enzyme complex. Molecular processes that pre-process DNA leading to its capture by Cas1–Cas2 are poorly understood but require DNA repair systems, including activities of RecBCD nuclease-helicase. Genetic analysis presented here challenges the current model that nuclease functions of RecBCD generate DNA that can be captured by Cas1–Cas2 (21). The genetic data show that *recB1080* and *recD recA* cells that lack RecBCD nuclease activity were proficient at acquiring new spacers, even in the face of plasmid instability associated with these *recBCD* genotypes. Removing RecA from *recD* cells unmasked the adaptation proficiency by removing the inhibitory effect of recombination on adaptation. Interestingly, *recB recA* cells acquired new spacers much less well than wild type cells, implicating an alternative activity of RecBCD in supporting naïve adaptation, most likely helicase function but in agreement that RecBCD is required in some way for naïve adaptation in *E. coli*.

RecBCD binds preferentially to duplex DNA ends (35,36), resects them into DNA fragments depending on prevailing buffer conditions (e.g. availability to the nuclease active site of metal ions and DNA) and on the translocation rate of helicase sub-units, but RecBCD helicase and nuclease activities are not dependent on one another (37,38). Helicase and nuclease functions are modulated when RecBCD encounters *Chi* DNA sequence, and together these events promote DNA repair by homologous recombination because they initiate RecA loading by RecBCD onto 3′ tailed ssDNA (39). However, the genetic data presented here suggest that functions of RecBCD in DNA repair by recombination are separate from how it promotes naïve adaptation: Critically, removal of RecA from cells, therefore removing the loading role of RecBCD in recombination, restored naïve adaptation.

**Figure 6.**
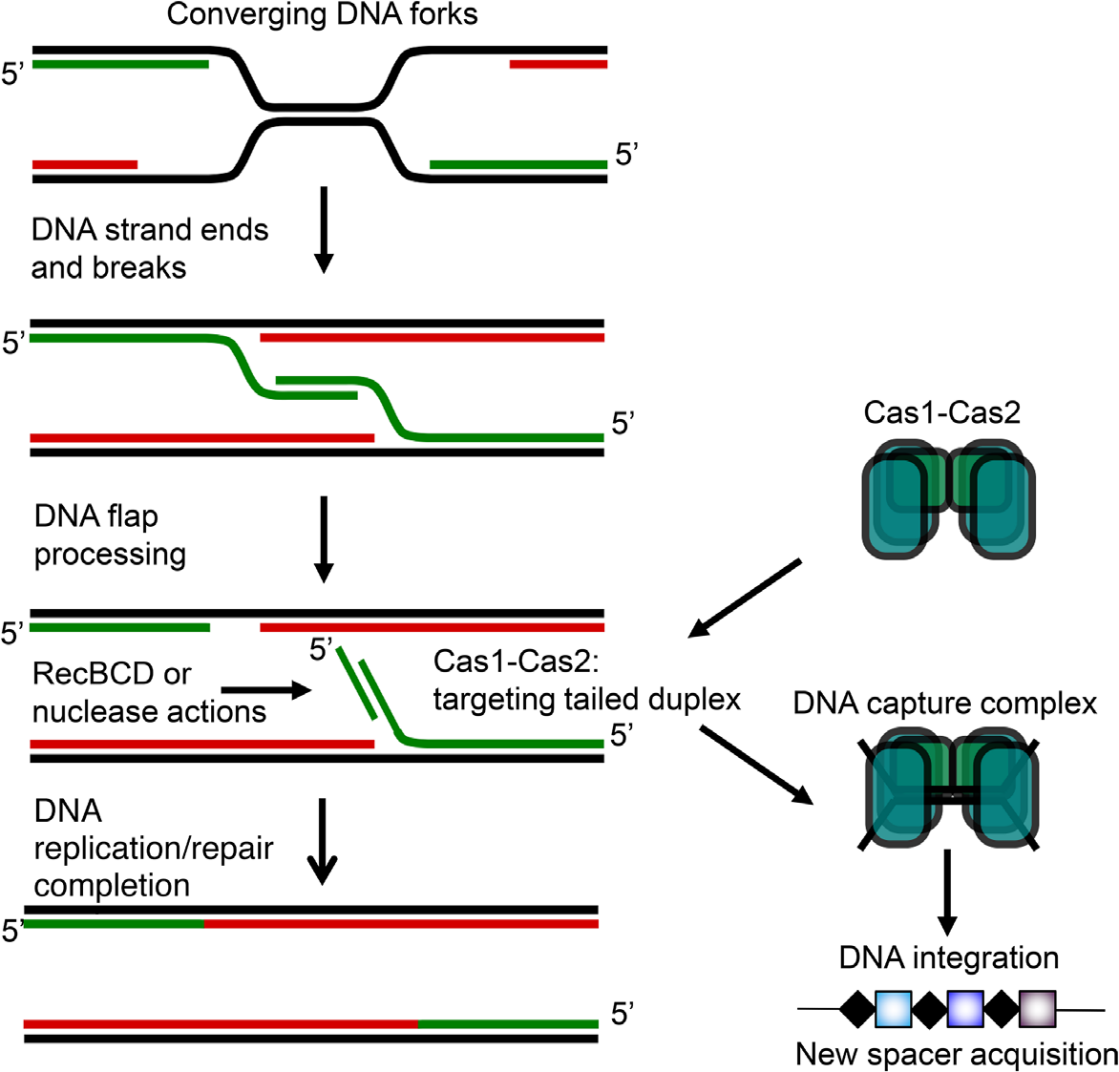
A model summarizing one way in which 5′ ssDNA tailed duplexes can arise in an area of the genome (*Ter* sites) that is targeted for new spacer acquisition during CRISPR–Cas adaptation reactions, and is processed by RecBCD and other enzymes during the normal cell cycle. The role of RecBCD during replication termination is unclear but its helicase activity may contribute to removal of nucleoprotein roadblocks in this context. Similar DNA structures that may be targeted by Cas1–Cas2 could also arise during global DNA and repair of replication forks, and during lagging strand replication of phage in the later stages of its replicative cycle.

It was significant that cells expressing *recB1080* (27) were adaptation proficient further indicating that RecBCD nuclease activity is not needed for naïve adaptation. RecB^1080^CD is a proficient helicase that translocates DNA with dual directionality 3′ to 5′ (RecB) and 5′ to 3′ (RecD) (40). The adaptation phenotypes associated with *recBCD* might indicate that DNA pre-processing and capture for naïve adaptation requires DNA translocation unwinding associated with RecB. RecBCD is a powerful translocase that can clear DNA of RNA polymerase, nucleosome and other DNA bound proteins (41,42). We propose that RecBCD helicase-translocase activities are required for adaptation to disrupt or displace nucleoprotein complexes present at DNA capture sites to provide access to DNA for Cas1–Cas2 and generate substrates that can be acted on by Cas1–Cas2 for DNA capture (Figure 6). We observed that the majority of new spacers were acquired from the *E. coli* chromosome compared to the Cas1–Cas2 plasmid whether in cells with fully functional RecBCD or in RecBCD compromised cells. This differs from a previous study (21) in which spacers were mainly derived from plasmid depending on whether or not Cas1–Cas2 protein expressed was induced or not. The previous study used BL-21AI strain (*E. coli* B) while we used *E. coli* K-12, which could be the reason for the observed difference. Another study (23) reported that *P. furiosus* cells acquired 96–99% of the unique spacers from the chromosome compared to 1–4% of new spacers derived from a plasmid expressing Cas proteins.

If RecBCD nuclease activity is not needed for naïve adaptation, how is DNA fragmented for capture? The genetic data presented here and in previous work suggest that neither 3′ ssDNA exonucleases nor 5′ ssDNA exonucleases have significant roles in DNA pre-processing for adaptation by Cas1–Cas2. Instead, we propose that Cas1–Cas2 nuclease activity when targeted to DNA end structures with PAM sequences can result in protospacer DNA capture prior to new spacer integration. Nuclease activity of *E. coli* Cas1 has been detected previously on a variety of model branched DNA substrates (20,43). The observation from genetics that deletion of 5′ ssDNA exonucleases in *recD recA* cells caused a significant improvement to naïve adaptation suggested that substrates for these enzymes (5′ ssDNA tailed DNA) may resemble those targeted by Cas1–Cas2. Purified Cas1–Cas2 complex was able to bind and nick these substrates without a requirement for PAM sequence, in this case AAC, being present. Although DNA structures present at DNA replication termini are not determined, broken replication forks processed at DNA ends by RecBCD for repair by recombination inhibit adaptation. If recombination is unable to occur because of mutations in RecBCD or RecA, or because Chi sequences are unavailable in foreign DNA then processing of DNA ends by alternative nucleases to RecBCD might promote Cas1–Cas2 activity at these sites, leading to DNA capture. Such an effect could explain the enrichment of new spacers acquired from replication termination sequences during naïve adaptation in *E. coli* (21). In *wt* cells, processing of broken replication forks involves asymmetric degradation of *ter*-oriented DNA ends (44) that may explain enrichment of new spacers from *ter* in these cells. In wild type cells Chi sequences place limits on spacer acquisition at *ter* regions (21) that seem to be released in *recBCD* mutants (Figure 1D). Structures of phage genomes during late rolling circle DNA replication form linear concatamers of DNA that include 5′ ssDNA tailed regions for lagging strand DNA synthesis. These may be important for targeting by Cas1–Cas2 for DNA capture as part of establishing CRISPR immunity to a newly encountered MGE. Similarly, events at DNA replication termination sites potentially generate DNA ends and 5′ ssDNA tailed DNA structures that are processed as part of the normal cell cycle by genome stability enzymes, including RecBCD (Figure 6). The 3′ to 5′ polarity of Cas3 DNA translocase activity would also generate 5′ ssDNA tailed DNA if it acts as a helicase, which may be important for DNA capture in the context of CRISPR interference reactions (45). Further work will be needed to determine the molecular mechanisms of DNA capture during adaptation, in particular using *in vitro* reactions with defined components that couple DNA replication, DNA repair and CRISPR adaptation.

## DATA AVAILABILITY

Updated DNA sequencing data for identifying newly acquired spacers is freely available from authors’ ResearchGate pages:

https://www.researchgate.net/profile/Ekaterina_Savitskaya

https://www.researchgate.net/profile/Edward_Bolt

https://www.researchgate.net/profile/Ivana_Ivancic_Bace

And is also available as supplementary material to this manuscript.

## Supplementary Material

Supplementary Data
