# Supplementary material for: CRISPR–Cas adaptation in Escherichia coli requires RecBCD helicase but not nuclease activity, is independent of homologous recombination, and is antagonized by 5′ ssDNA exonucleases
Source: Nucleic Acids Res. 2018 Sep 5;46(19):10173–83. doi: 10.1093/nar/gky799 (PMC6212769; doi:10.1093/nar/gky799)
Supplement: Supplementary Data [file gky799_supplemental_files.zip › supp data and figures.pdf]

Supplementary Data

Table S1. *E. coli* strains used in this study listed below were derived from BW25113.

Bacterial strain	Relevant genotype	Source or reference
MG1655	+ F^- *rec*$^+$	Bachmann 1996
SLM1023	+ *xseA*::*dhfr*	C. Rudolph
IIB1151	+ *recD1903*::mini-Tn*10*	Laboratory collection
AM1986	+ Δ*recA1921*::*spec*	C. Rudolph
TH446	+ *recA*::*cam*	Laboratory collection
IIB360	+ *recB1080 argA*::Tn*10*	Ivančić-Baće et al. 2006
LMM1032	+ *recJ2052*::Tn*10kan*	D. Zahradka
LMM1247	+ *sbcD*::*kan*	D. Zahradka
N5288	+ *exoX1*::*npt*	C. Rudolph
JW1993-1	+ *sbcB780*::*kan*	D. Zahradka
BW25113	Δ(*araD-araB*)*567* Δ*lacZ4787*(::*rrnB-3*) $λ^-$ *rph*-1, Δ(*rhaD-rhaB*)*568 hsdR514*	Wanner BL
	Strains related to BW25113	
IIB892	+ Δ*cas3*::*apra* Δ*casC760*::*kan*^{S}	Ivančić-Baće et al. 2015
BW39183	+ Δ*cas1*::*kan*	Keio collection, E. Semenova
IIB1156	+ Δ*cas1*::*kan* Δ*cas3*::*apra* Δ*casC760*::FRT	P1. IIB892 x BW39183
IIB1157	+ Δ*cas1*::*kan* Δ*cas3*::*apra* Δ*casC760*::FRT *recD1903*::mini-Tn*10*	P1. IIB1151 x IIB1156
IIB1165	+ Δ*cas3*::*apra* Δ(*casC-cas1*::FRT)	Removal of kan by pCP20
IIB1192	+ Δ*cas3*::*apra* Δ(*casC-cas1*::FRT) *recD1903*::mini-Tn*10*	P1. IIB1151 x IIB1157
IIB1195	+ Δ*cas3*::*apra* Δ(*casC-cas1*::FRT) *recD1903*::mini-Tn*10 recJ2052*::Tn*10kan*	P1. LMM1032 x IIB1192
IIB1199	+ Δ*cas3*::*apra* Δ(*casC-cas1*::FRT) *recJ2052*::Tn*10kan*	P1. LMM1032 x IIB1165
IIB1207	+ Δ*cas3*::*apra* Δ(*casC-cas1*::FRT) *recD1903*::mini-Tn*10 sbcD*::*kan*	P1. LMM1247 x IIB1192
IIB1208	+ Δ*cas3*::*apra* Δ(*casC-cas1*::FRT) *recB268*::Tn*10*	P1. N3071 x IIB1165
IIB1211	+ Δ*cas3*::*apra* Δ(*casC-cas1*::FRT) *recD1903*::mini-Tn*10 xseA*::*dhfr*	P1. SLM1023 x IIB1192
IIB1213	+ Δ*cas3*::*apra* Δ(*casC-cas1*::FRT) *recD1903*::mini-Tn*10 sbcD*::*kan recA*::*cam*	P1. TH446 x IIB1207
IIB1214	+ Δ*cas3*::*apra* Δ(*casC-cas1*::FRT) *recB1080 argA*::Tn*10*	P1. IIB360 x IIB1165
IIB1215	+ Δ*cas3*::*apra* Δ(*casC-cas1*::FRT) *recD1903*::mini-Tn*10 exoX1*::*npt*	P1. N5288 x IIB1192
IIB1218	+ Δ*cas3*::*apra* Δ(*casC-cas1*::FRT) *recD1903*::mini-Tn*10 xseA*::*dhfr recA*::*cam*	P1. TH446 x IIB1211
IIB1221	+ Δ*cas3*::*apra* Δ(*casC-cas1*::FRT) *recB1080 argA*::Tn*10 recA*::*cam*	P1. TH446 x IIB1214
IIB1222	+ Δ*cas3*::*apra* Δ(*casC-cas1*::FRT) *recD1903*::mini-Tn*10 exoX1*::*npt recA*::*cam*	P1. TH446 x IIB1215
IIB1226	+ Δ*cas3*::*apra* Δ(*casC-cas1*::FRT) *xseA*::*dhfr*	P1. SLM1023 x IIB1165
IIB1227	+ Δ*cas3*::*apra* Δ(*casC-cas1*::FRT) *recD1903*::mini-Tn*10 xseA*::*dhfr*	P1. LMM1032 x IIB1211

	recJ2052::Tn*10kan*	
IIB1228	+ Δ*cas3*::*apra* Δ(*casC-cas1*::FRT) *sbcD*::*kan*	P1. LMM1247 x IIB1165
IIB1229	+ Δ*cas3*::*apra* Δ(*casC-cas1*::FRT) *exoX1*::*npt*	P1. N5288 x IIB1165
IIB1235	+ Δ*cas3*::*apra* Δ(*casC-cas1*::FRT) *xseA*::*dhfr* *recJ2052*::Tn*10kan*	P1. LMM1032 x IIB1227, Tc^S
IIB1136	+ Δ*cas3*::*apra* Δ(*casC-cas1*::FRT) *recD1903*::mini-Tn*10* *xseA*::*dhfr* *recJ2052*::Tn*10kan* *recA*::*cam*	P1. TH446 x IIB1227
IIB1239	+ Δ*cas3*::*apra* Δ(*casC-cas1*::FRT) *sbcB780*::*kan*	P1. JW1993-1 x IIB1165
IIB1240	+ Δ*cas3*::*apra* Δ(*casC-cas1*::FRT) *recD1903*::mini-Tn*10* *sbcB780*::*kan*	P1. JW1993-1 x IIB1192
IIB1242	+ Δ*cas3*::*apra* Δ(*casC-cas1*::FRT) *recD1903*::mini-Tn*10* *sbcB780*::*kan* *recA*::*cam*	P1. TH446 x IIB1240
IIB1244	+ Δ*cas3*::*apra* Δ(*casC-cas1*::FRT) *recB268*::Tn*10* *recA*::*cam*	P1. TH446 x IIB1242
IIB1245	+ Δ*cas3*::*apra* Δ(*casC-cas1*::FRT) *recD1903*::mini-Tn*10* *recA*::*cam*	P1. TH446 x IIB1192
IIB1248	+ Δ*cas3*::*apra* Δ(*casC-cas1*::FRT) *xseA*::*dhfr* *recJ2052*::Tn*10kan* *recA*::*cam*	P1. TH446 x IIB1235
IIB1252	+ Δ*cas3*::*apra* Δ(*casC-cas1*::FRT) *recA*::*cam*	P1. TH446 x IIB1165
IIB1253	+ Δ*cas3*::*apra* Δ(*casC-cas1*::FRT) *xseA*::*dhfr* *recA*::*cam*	P1. TH446 x IIB1226
IIB1254	+ Δ*cas3*::*apra* Δ(*casC-cas1*::FRT) *exoX1*::*npt* *recA*::*cam*	P1. TH446 x IIB1229
IIB1255	+ Δ*cas3*::*apra* Δ(*casC-cas1*::FRT) *sbcB780*::*kan* *recA*::*cam*	P1. TH446 x IIB1239
IIB1258	+ Δ*cas3*::*apra* Δ(*casC-cas1*::FRT) *sbcD*::*kan* *recA*::*cam*	P1. TH446 x IIB1228
IIB1259	+ Δ*cas3*::*apra* Δ(*casC-cas1*::FRT) *recJ2052*::Tn*10kan* *recA*::*cam*	P1. TH446 x IIB1199

Genotypes of newly created strains were confirmed by PCR using the following primers:

ygcJ-3: 5'-GGATGTTGACCTGGTGG

ygcJ-4: 5'-GCACACTCTCTGATAACG

cas1del-F: 5' CAGCTAAATCGATGGGATGTG 3'

cas1del-R: 5’ GATGGCTAATCTGCCTCGTAAG 3’

apra 1 (R): 5’ CCA GAA TGT GTC AGA GAC AAC 3’

upcas3 (F): 5’ CGA TAT TTA TGA GCA GCA TC 3’

The *sbcD* mutation was verified by comparing the plaqing efficiencies of mutant λ*pal* phage on *wt* and *sbcCD* mutants.

Table S2.

Strain (passage)	% of spacer acquisition +/- standard deviation from the mean
IIB1165 (*wt*) + pBad empty plasmid (3rd)	0.00: control used to give baseline zero reading.
IIB1165 (*wt*) + pEB628 (3rd)	47.9 +/- 10.7
IIB1192 (*recD*) + pEB628 (3rd)	3.0 +/- 0.5
IIB1208 (*recB*) + pEB628 (2nd)	6.0 +/- 4.0
IIB1208 (*recB*) + pEB628 (3rd)	1.0 +/- 1.7
IIB1244 (*recB recA*) + pEB628 (2nd)	18.5 +/- 3.9

Naïve adaptation in strains *recD*, *recB* and *recB recA* was not readily detectable in agarose gels of PCR across CRISPR-1 in data presented in Figure 1. Gel areas corresponding to gel “blank space”, where new spacer DNA would be expected to migrate if present but undetectable by that method, was extracted and used as a template for a further iteration of PCR using primers annealing to spacer 3 and leader-repeat 1 border:

CRISPR-NGS-F: 5’-TGCTTTAAGAACAAATGTATACTTT-3'

CRISPR-NGS-R: 5'-CAACATTATCAATTACAACCGA-3'

Outcomes from this second PCR are a 217 bp DNA product for no new spacer detection or 278 bp if new spacer was detectable. Percentage of any detectable spacer acquisition was obtained by measuring relative band intensities using Kodak 1D Image Analysis Software v. 3.6.0. Wild type strain transformed with pEB628 was used as a positive control.

Table S3. Efficiency of spacer acquisition expressed as relative band intensity; data is for strains in which new spacer acquistion was detectable at greater than zero.

**Strain (+ pCas1-Cas2)**	**Genotype**	**Percentage of spacer acquisition (%)**		
		**1 p**	**2 p**	**3 p**
IIB1165	*wt*	2.2 ± 0.2	8.8 ± 3	11 ± 1.5
IIB1252	*recA*	3.1 ± 0.7	8.6 ± 0.8	13.3 ± 1.1
IIB1245	*recD recA*	4 ± 2.4	9.1 ± 0.6	2.9 ± 0.3
IIB1214	*recB1080*	2.4 ± 0.02	0.68 ± 0.2	0.77 ± 0.1
IIB1239	*sbcB*	0.27 ± 0.05	13.6 ± 1.9	14.9 ± 1.9
IIB1226	*xseA*	0.25 ± 0.2	12.5 ± 3.8	19.1 ± 6.2
IIB1228	*sbcD*	1.4 ± 0.4	8.4 ± 3.8	17.2 ± 6.5
IIB1229	*exoX*	1.6 ± 1.1	8.6 ± 3.5	16.7 ± 4.8
IIB1253	*xseA recA*	3.73 ± 0.5	12.69 ± 1	19.3 ± 2.4
IIB1254	*exoX recA*	4.7 ± 1.7	13.7 ± 0.7	23.4 ± 3
IIB1255	*sbcB recA*	5.6 ± 3.4	11.1 ± 1.8	8.3 ± 1.8
IIB1258	*sbcD recA*	5 ± 0.1	16.2 ± 0.4	21.7 ± 0.05
IIB1242	*recD sbcB recA*	1.56 ± 0.3	0.78 ± 0.4	0.32 ± 0.3
IIB1218	*recD xseA recA*	3.9 ± 4.2	10.02 ± 1.3	2.59 ± 0.6
IIB1222	*recD exoX recA*	3.1 ± 0.4	1.59 ± 0.4	1.2 ± 0.6
IIB1213	*recD sbcD recA*	2.28 ± 2	0.3 ± 0.19	0.28 ± 0.1
IIB1199	*recJ*	0.7 ± 0.47	10.2 ± 2.5	20.5 ± 1.5
IIB1235	*xseA recJ*	2.1 ± 0.5	4.3 ± 3	7.1 ± 2.8
IIB1246	*xseA sbcD*	1.8 ± 1.5	10.8 ± 2.2	15.9 ± 6.4
IIB1247	*xseA exoX*	0.7 ± 0.1	7.7 ± 1.1	13.6 ± 3.3
IIB1259	*recJ recA*	2.8 ± 0.4	13.9 ± 0.2	18.5 ± 2
IIB1209	*recD recJ recA*	3.29 ± 1.3	1.7 ± 1.4	1.5 ± 0.88

IIB1223	*recD xseA exoX recA*	2.7 ± 0.02	6.8 ± 3	0.75 ± 0.5
IIB1224	*recD xseA sbcD recA*	3.27 ± 0.5	6.48 ± 0.37	0.73 ± 0.5
IIB1236	*recD xseA recJ recA*	8 ± 2	16.3 ± 2	19.2 ± 6
IIB1248	*xseA recJ recA*	3.1 ± 0.6	5.9 ± 1.4	9.7 ± 0.7
IIB1256	*xseA sbcD recA*	1.8 ± 1.5	12.8 ± 7.3	19.6 ± 5.6
IIB1257	*xseA exoX recA*	3.8 ± 2.2	14.1 ± 1	20.3 ± 4.2

Plasmid stability measurements and adaptation

To better understand if poor naïve adaptation, which is stimulated by overexpression of Cas1-Cas2 from the plasmid, in certain mutants was caused by plasmid instability than mutation(s) itself (i.e. *recB* or *recD*) we determined the number of cells that kept the plasmid after three sub-cultivations (passages). Indeed, we noted that adaptation experiments were strongly influenced by the stability of the Cas1-Cas2 expressing plasmid (pEB628) in cells. Mutants of *recB* or *recD* are known for plasmid instabilities and defects in replication termination (Wendel et al. 2014). Overall, Cas1-Cas2 expressing plasmid was stable in *wt* and *recA* cells, moderately lost in *recD* (about 500-fold) and ≈ 10^3 fold in *recB* and completely lost in *recD recJ* cells after two sub-cultivations (data not shown). A similarly strong effect was also noticed in *recD exoX*, *recD sbcD*, *recD sbcB* and *recD xseA recJ* cells (data not shown), strains that have longer ssDNA tails that probably provoke recombination and generate plasmid multimers that are eventually removed from cells.

Table S4. Plasmid instability measurements of major strains referred to in the results measured during passage two during naive adaptation assays

**Strain**	**Genotype**	**Number of cells containing**<br>**pBad**	 **pEB628**
		**(x 10^7)**	
IIB1165	*wt*	233 ± 15	193 ± 10
IIB1208	*recB*	88 ± 25	0.1 ± 0.08
IIB1192	*recD*	17 ± 15	0.4 ± 0.3

IIB1252	*recA*	133 ± 18	13 ± 0.5
IIB1245	*recD recA*	47 ± 3	0.67 ± 0.46
IIB1244	*recB recA*	55 ± 28	0.008 ± 0.005
IIB1214	*recB1080*	55 ± 7	0.08 ± 0.08
IIB1221	*recB1080 recA*	18 ± 5	0.008 ± 0.002
IIB1242	*recD sbcB recA*	45 ± 12	-
IIB1218	*recD xseA recA*	61 ± 20	0.4 ± 0.3
IIB1222	*recD exoX recA*	17 ± 15	0.016 ± 0.02
IIB1213	*recD sbcD recA*	45 ± 14	-
IIB1253	*xseA recA*	55 ± 7	5.7 ± 1
IIB1254	*exoX recA*	57 ± 25	7 ± 2.4
IIB1255	*sbcB recA*	52 ± 10	0.65 ± 0.4
IIB1258	*sbcD recA*	85 ± 28	5.5 ± 2.3

Supplementary Figures and methods

Figure S1

(A). Elution profile of Cas1-Cas2 co-purification by Superdex S200 gel filtration showing Cas1-Cas2 complex formation and its separation from Cas1 alone, visualised by coomassie staining of fractions analysed by SDS-PAGE. **(B).** *In vitro* assay to detect new spacer integration (“spIN”) into the *E. coli* CRISPR-1 DNA sequence (25 nM) catalysed by purified *E. coli* Cas1-Cas2 complex (250 nM). This reaction is optimised for integration by using a synthetic DNA protospacer made from annealing two ssDNA oligonucleotides of sequences described in (Nunez et al. 2015) and by adding purified *E. coli* Integrase Host Factor (IHF, 250 nM). CRISPR-1 DNA for integration comprised the leader and first two spacer-repeat pairs of the CRISPR-1 locus from *E. coli* MG1655. CRISPR-1 was generated with a 5’ Cy5 end-label by PCR from CRISPR-1 cloned into pUC18 generating a plasmid (pJRW2), using the method described below.

IHF protein was made as described in the main results. PCR amplification of the genes encoding each IHF subunit used the following primers:

IHFα forward
5’-
ACGTCGGATCCGGAAAACCTGTATTTTCAGGGCTCCATGGCGCTTACAAAAGCTGAAAT
GTC
IHFα reverse
5’ ACGTCGCGGCCGCTTACTCGTCTTTGGGCGAAGCG

IHFβ forward

5’ ACGTCCTCGAGACCAAGTCAGAATTGATAGAAAGACTTGCC

IHFβ reverse

5’ ACGTCCCTAGGTTAACCGTAAATATTGGCGCGATCGC

Cy-5 end labeled CRISPR-1 DNA for spacer integration (spIN) assays was generated by PCR of CRISPR-1 from *E. coli* MG1655 cloned into pUC19 (pJRW2) using the following primers:

Crispr1 F ; 5’ Cy5-AGAATTAGCTGATCTTTAATAATAAGG and Crispr1 R short;

5’ TCTCAACATTATCAATTACAACCG

The PCR reaction contained 1 ng of pJRW2 and the following reagents in a final volume of 50 μl using Vent DNA polymerase (NEB). PCR reactions were as follows:

95 ^{o}C – 5 min, followed by thirty cycles of 95 ^{o}C – 30 sec.; 71 ^{o}C – 30 sec.; 72 ^{o}C – 30 sec. And finally 72 ^{o}C for 5 min. CRISPR-1 DNA product (284 base pairs) was purified using gel extraction kits (QiaGen) and verified for size in an agarose gel:

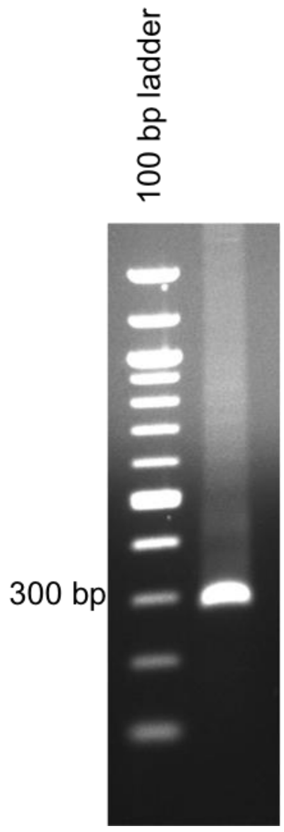

The full sequence of CRISPR-1 DNA that was amplified for use in spIN reactions is:

AGAATTAGCTGATCTTTAATAATAAGGAAATGTTACATTAAGGTTGGTGGGTTGTTTTTAT
GGGAAAAAATGCTTTAAGAACAAATGTATACTTTTAGAGAGTTCCCCGCGCCAGCGGGG
ATAAACCGCTTTCGCAGACGCGCGGCGATACGCTCACGCAGAGTTCCCCGCGCCAGC

GGGGATAAACCGCAGCCGAAGCCAAAGGTGATGCCGAACACGCTGAGTTCCCCGCGCCAGCGGGGATAAACCGGGCTCCCTGTCGGTTGTAATTGATAATGTTGAGA

Figure S2

Summary of DNA substrates used in this work.

Figure S3

(A). Histogram showing the ratio between numbers of plasmid (P) and chromosomal (C) dervied new spacers. (B and C). Agarose gels summarizing lack of CRISPR expansion in the *E. coli* strains lacking *recD* and ssDNA exonucleases indicated in three passages (p1 – p3) in all cases containing plasmid encoding inducible Cas1-Cas2 (pCas1-2).

Figure S4

EMSA showing Cas1-Cas2 complex formation and aggregation when mixed with an optimized protospacer DNA substrate that is used widely for analyzing Cas1-Cas2 catalyzed spacer integration into CRISPR DNA sequences (Nunez *et al* 2015). Cy5 end labeled DNA substrate (20 nM, see also Supplementary Figure S2) was incubated with 0, 31.25, 62.5, 125, 250, 500 nM Cas1-Cas2 complex for 30 minutes at 37 ^{o}C followed by analysis on a 5 % native acrylamide gel and imaged using a FLA3000 (FujiFilm).

Figure S5

Denaturing (urea) gel analysis of DNA cutting of DNA-10 and a the equivalent 3' ssDNA tailed duplex (DNA-3') by Cas1-Cas2 complex or Cas1 alone. Proteins were used at 0, 31.25, 62.5, 125, 250 and 500 nM. Reactions were carried out at 37°C for 60 minutes. Gel analysis was by 15 % acrylamide urea (8 M) denaturing gels that were imaged for Cy5 imaged using a FLA3000 (FujiFilm).

Figure S6

Denaturing (urea) gel showing in summary the cutting of DNA-10 (lanes 2 - 4) and DNA-11 (lanes 6 – 8) and its use to determine cut sites. Marker DNA lengths in nucleotides are shown alongside three independent reactions mixing Cas1-Cas2 (250 nM) with DNA (20 nM) as indicated. To determine the cut sites the migration distance of DNA bands from the marker DNA and cut products was measured from the base of the well. Migration distance (mm) of marker oligonucleotides was plotted against nucleotide length (Log10) using Prism (GraphPad software) with nuclease product size interpolated from the graph.

Supplementary Reference

Nunez, J. K., et al. (2015), 'Integrase-mediated spacer acquisition during CRISPR-Cas adaptive immunity', *Nature,* 519 (7542), 193-8.

Figure S1

A.

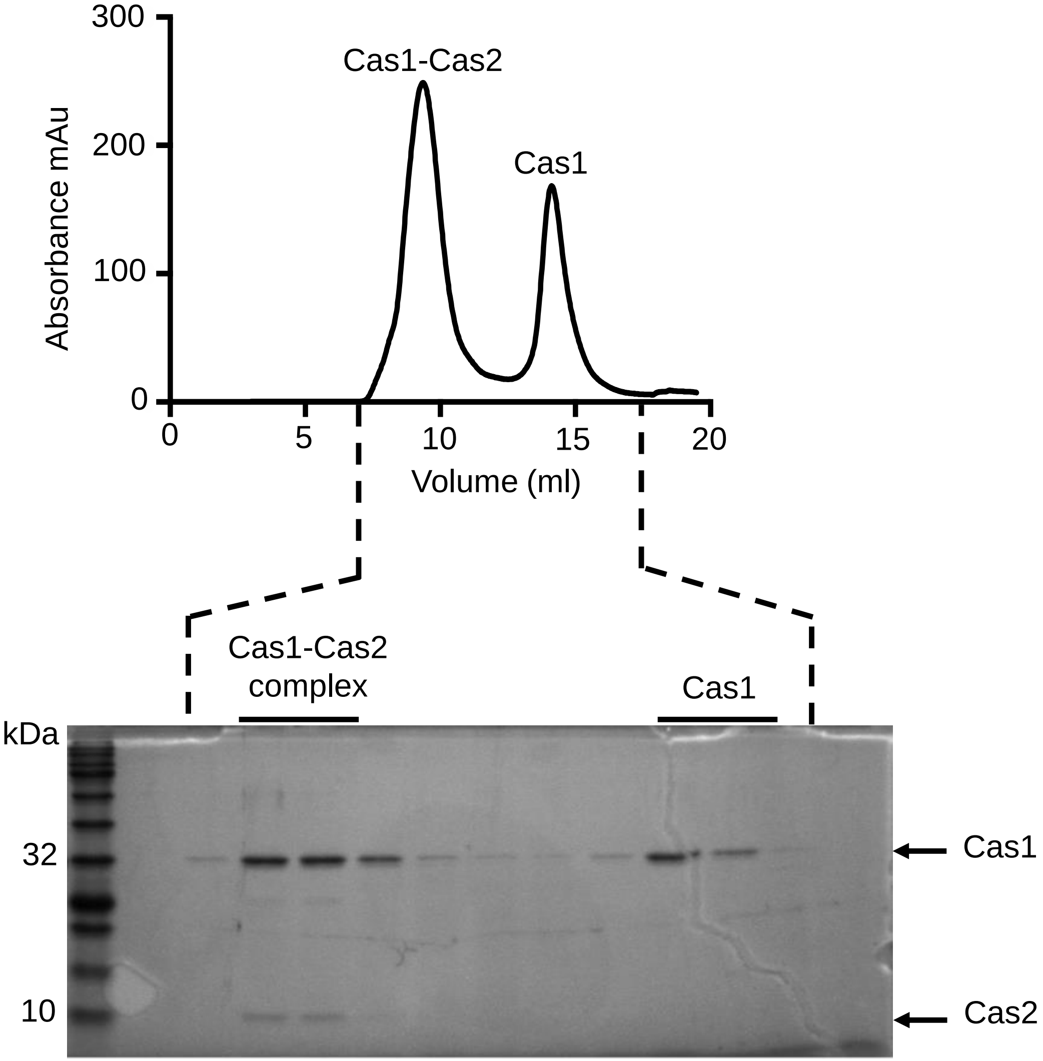

300
200
100
0
Absorbance mAu
Cas1-Cas2
Cas1
0
5
10
15
20
Volume (ml)
Cas1-Cas2
complex
Cas1
kDa
32
10
Cas1
Cas2

B.

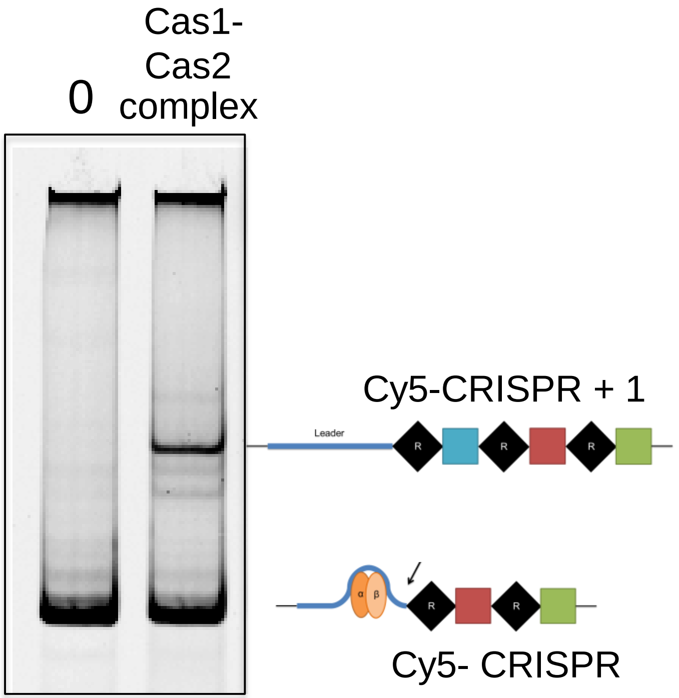

0
Cas1-
Cas2
complex
Cy5-CRISPR + 1
Leader
R
R
R
Cy5- CRISPR

Figure S2

```
DNA-10          CGGACGAACAACGAACAATGCCAACTAAAGCTCAACCCAGGTGAAACTAA-Cy5 5’
              5’GCCTGCTTGT

DNA-11          CGGACGAACAACGAACAATGCCAACTAAAGCTCAACCCAGGTGAAACTAA-Cy5 5’
              5’GCCTGCTTGTT

DNA-12          CGGACGAACAACGAACAATGCCAACTAAAGCTCAACCCAGGTGAAACTAA-Cy5 5’
              5’GCCTGCTTGTTG

DNA-13          CGGACGAACAACGAACAATGCCAACTAAAGCTCAACCCAGGTGAAACTAA-Cy5 5’
              5’GCCTGCTTGTTGC

DNA-14          CGGACGAACAACGAACAATGCCAACTAAAGCTCAACCCAGGTGAAACTAA-Cy5 5’
              5’GCCTGCTTGTTGCT

DNA-15          CGGACGAACAACGAACAATGCCAACTAAAGCTCAACCCAGGTGAAACTAA-Cy5 5’
              5’GCCTGCTTGTTGCTT

DNA-14-TTT      CGGACGAACAACGTTTAATGCCTTTTAAAGCTCAACCCAGGTGAAACTAA-Cy5 5’
              5’GCCTGCTTGTTGCT

Duplex          CGGACGAACAACGAACAATGCCAACTAAAGCTCAACCCAGGTGAAACTAA-Cy5 5’
             5’ GCCTGCTTGTTGCTTGTTACGGTTGATTTCGAGTTGGGTCCACTTTGATT

DNA-3’          AATCAAAGTGGACCCAACTCGAAATCAACCGTAACAAGCAACAAGCAGGC-Cy5 5’
                                                      5’TGTTCGTCCG

Protospacer     TGCTCGCATCGACTCCGCTCCCCTGACG-Cy5 5’
                   5’CGTAGCTGAGGCGAGGGGACTGCTGGGC
```

Figure S3

A.

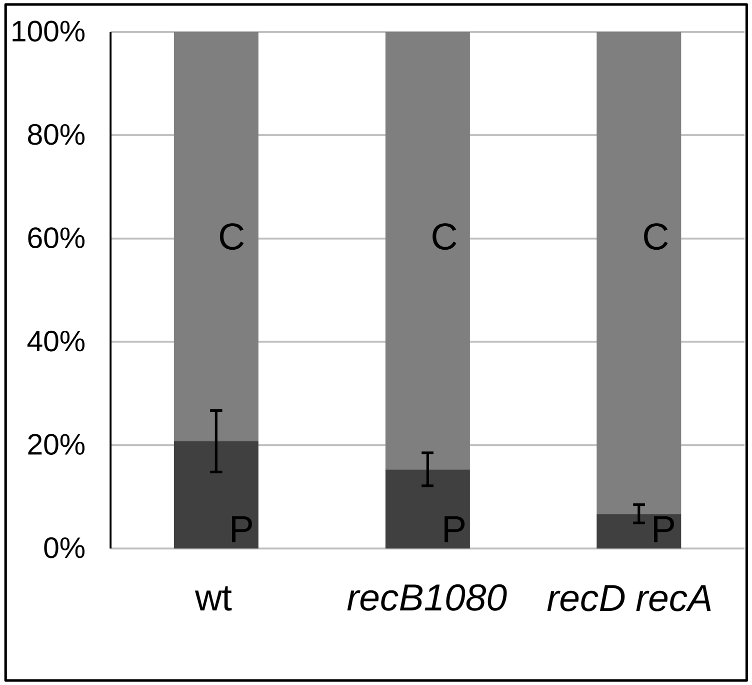

B.

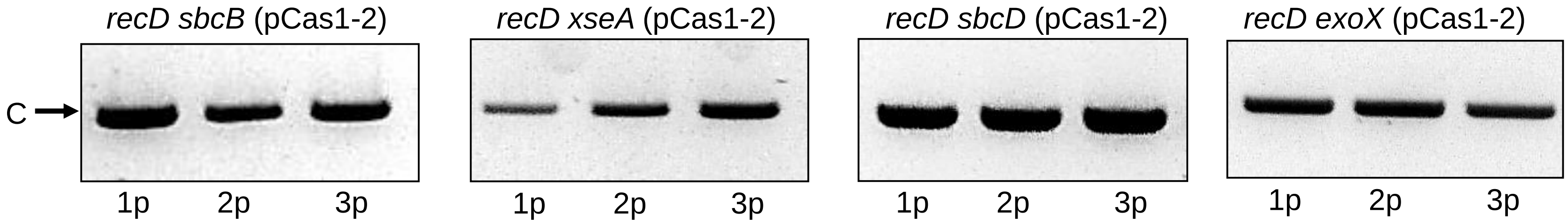

C.

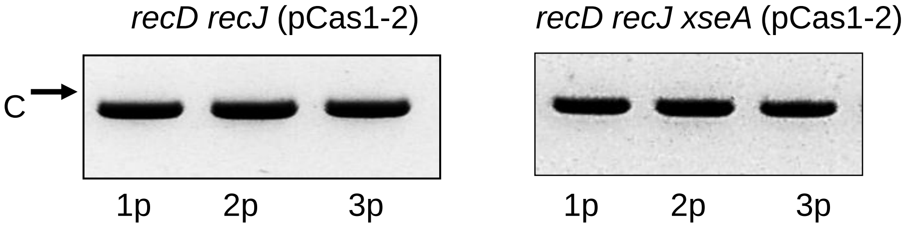

Figure S4

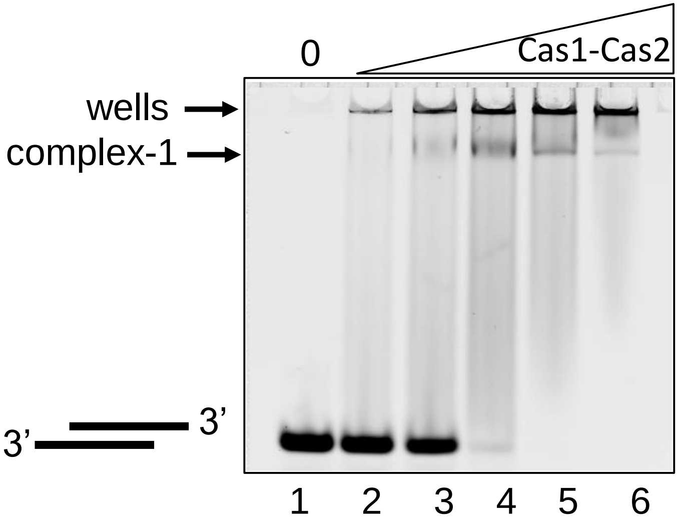

Figure S5

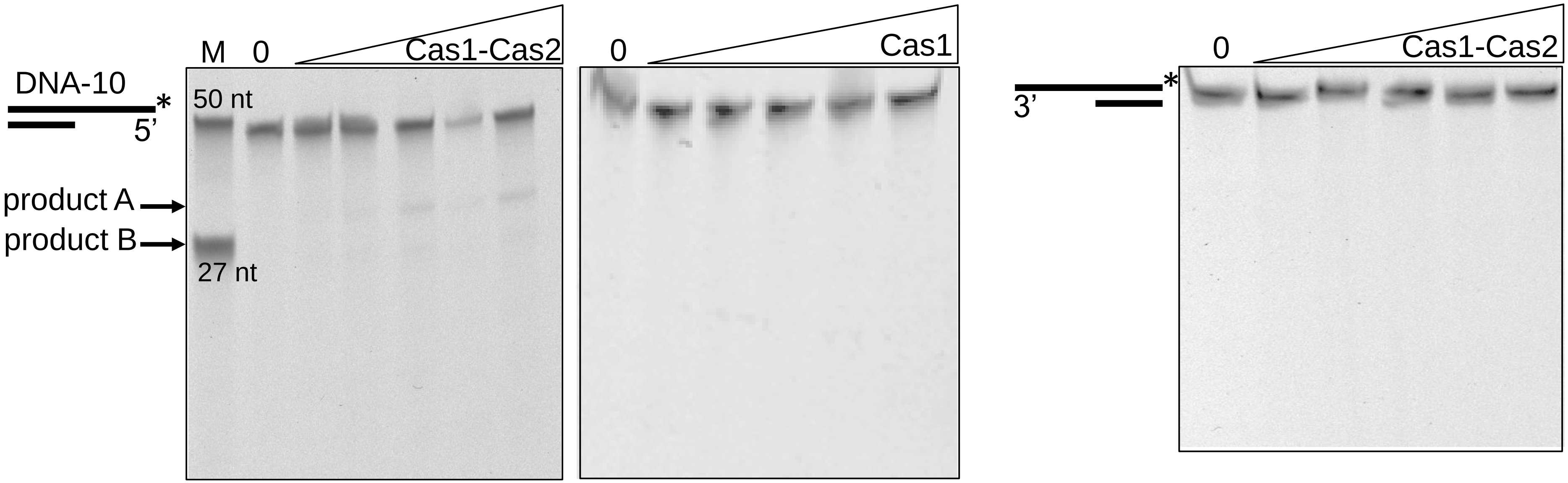

M
0
Cas1-Cas2
0
Cas1
0
Cas1-Cas2
DNA-10
*
5'
50 nt
product A
product B
27 nt
*
3'

Figure S6

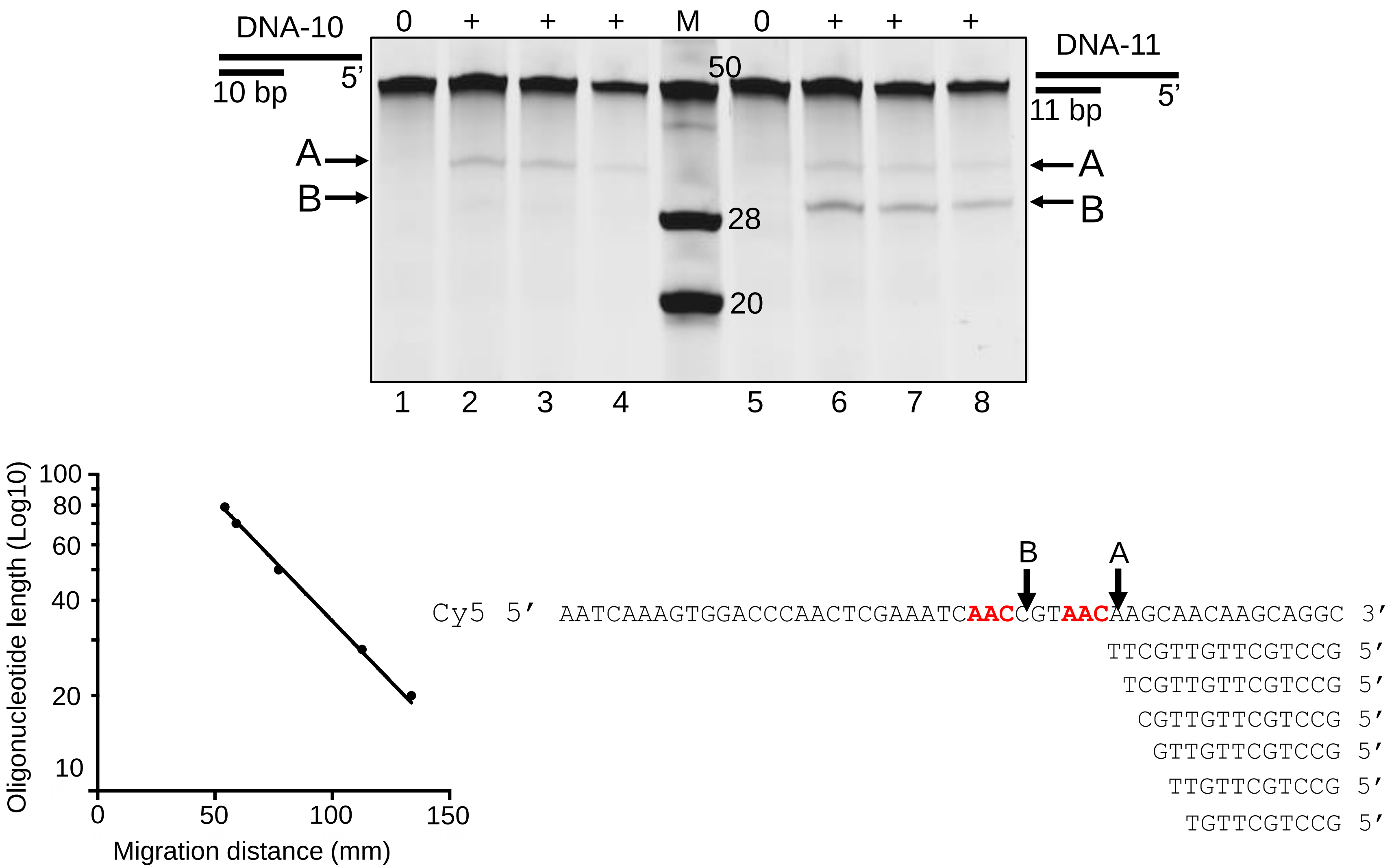

DNA-10
10 bp
5’
A
B
0 + + + M 0 + + +
50
28
20
DNA-11
11 bp
5’
A
B
1 2 3 4 5 6 7 8
Oligonucleotide length (Log10)
100
80
60
40
20
10
0 50 100 150
Migration distance (mm)
B A
Cy5 5’ AATCAAAGTGGACCCAACTCGAAATCAACCGTAACAAGCAACAAGCAGGC 3’
TTCGTTGTTCGTCCG 5’
TCGTTGTTCGTCCG 5’
CGTTGTTCGTCCG 5’
GTTGTTCGTCCG 5’
TTGTTCGTCCG 5’
TGTTCGTCCG 5’
